# Dynamic Interfacial Charge Regulation for High‐Fidelity Broadband Circularly Polarized Light Detection

**DOI:** 10.1002/advs.77641

**Published:** 2026-09-11

**Authors:** Siqin Zhou, Honghan Ji, Houchao Jing, Xue Jin, Ying Zhao, Pengfei Duan

**Affiliations:** ^1^ Laboratory of Nanosystem and Hierarchical Fabrication National Center for Nanoscience and Technology Beijing P. R. China; ^2^ University of Chinese Academy of Sciences Beijing P. R. China; ^3^ CHN Energy National Institute of Clean‐and‐Low‐Carbon Energy Beijing P. R. China

**Keywords:** chiral photonic films, circularly polarized light detection, dynamic interfacial charge regulation, photoelectrochemical transducers

## Abstract

Circularly polarized light (CPL) carries both chirality and polarization‐state information, offering unique opportunities for optical communication, polarization imaging, and three‐dimensional displays. However, high‐fidelity CPL detection is often limited to relatively narrow spectral ranges, while weak chirality‐dependent photocurrent can be readily obscured by dark current and noise during interface‐dependent solid‐state interfacial charge transport. Here, a broadband photoelectrochemical‐type CPL detection platform is developed by coupling a multi‐photonic‐bandgap chiral polymer modulation layer with a dynamically regulated BiVO_4_‐RGO‐MXene transducer. The stacked chiral polymer films impart wavelength‐ and chiral‐selective optical modulation ranging from 360 to 830 nm, while the adaptive photoelectrochemical interface enables efficient conversion of the encoded optical differences into electrical outputs through dynamic interfacial charge regulation. Consequently, broadband CPL inputs are selectively gated and translated into stable electrical signals within a single compact device. This architecture combines broadband chiral optical modulation with adaptive interfacial charge transduction, alleviating trap‐state interference and suppressing noise without increasing structural complexity. The present strategy provides a general route toward compact, broadband, and integration‐compatible CPL photodetection. Furthermore, CNN‐assisted recognition of CPL‐encrypted images demonstrates the capability of the device for noise‐robust and machine‐readable chiroptical information decoding.

## Graphical abstract

A photoelectrochemical circularly polarized light detector combines a multilayer chiral photonic film with a dynamically regulated BiVO_4_‐RGO‐MXene transducer. Broadband optical modulation from 360 to 830 nm is converted into stable electrical outputs through low‐noise solid‐liquid interfacial charge transfer, enabling self‐powered CPL discrimination with high fidelity, fast response, and robust operational stability.

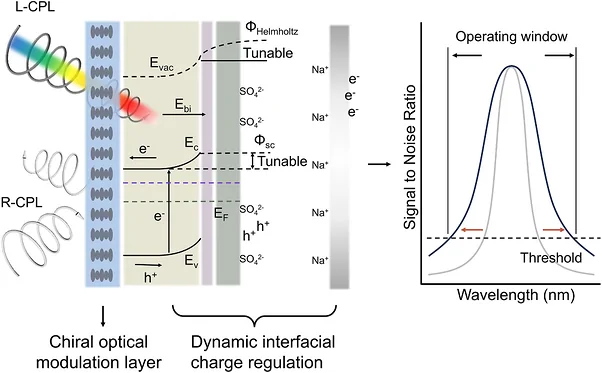

## 1 Introduction

Circularly polarized light (CPL) carries chirality and polarization‐state information, which plays a key role in quantum communication, polarization imaging, and three‐dimensional displays [1, 2, 3, 4]. Conventional CPL discrimination typically relies on wavelength‐dependent external quarter‐wave plates and linear polarizers, which increase system volume and complexity, thereby hindering miniaturization, chip‐level integration, and broadband operation [1, 5, 6, 7]. Therefore, developing a broadband, high‐performance, cost‐effective, and compact CPL framework is highly desirable [8].

Up to now, most CPL photodetectors adopt solid‐state architectures, in which optical chirality is encoded either within the photoactive material or externally through a chiral photonic layer coupled to an achiral semiconductor transducer [9, 10, 11, 12]. Therefore, high‐fidelity CPL detection is typically confined to a limited favorable spectral range determined by either the bandgap of the semiconductor or the photonic bandgap of the chiral layer [2, 13, 14]. Beyond this range, the weakened chirality‐dependent photocurrent becomes increasingly vulnerable to dark current and noise [15, 16, 17]. For intrinsically chiral photoactive materials, CPL discrimination arises directly from the chiral optical or spin‐dependent response of the active semiconductor and does not necessarily rely on a separate chiral/semiconductor interface. In contrast, for interface‐dependent architectures such as heterojunction devices or photonic‐layer/semiconductor systems, electrical readout additionally involves carrier transport across solid‐solid interfaces [18, 19, 20, 21]. Particularly in crystalline heterojunctions, interfacial lattice mismatch and crystallographic defects can introduce trap states and Fermi‐level pinning, thereby constraining interfacial charge distribution at static solid‐state junctions, limiting their adaptability to photoinduced carrier redistribution, and increasing dark current and noise while suppressing charge‐collection efficiency [22, 23, 24, 25]. As a result, the weak chirality‐dependent signal, particularly outside the favorable spectral range, is further amplified in its susceptibility to noise during interfacial charge transport, thereby severely limiting signal‐to‐noise performance and high‐fidelity readout [22, 26, 27, 28]. Therefore, for interface‐dependent CPL architectures, developing a broadband CPL photodetector that enables adaptive regulation of interfacial charges to address the limitations of static solid‐solid interfaces remains a significant challenge [29, 30, 31, 32].

**FIGURE 1:**
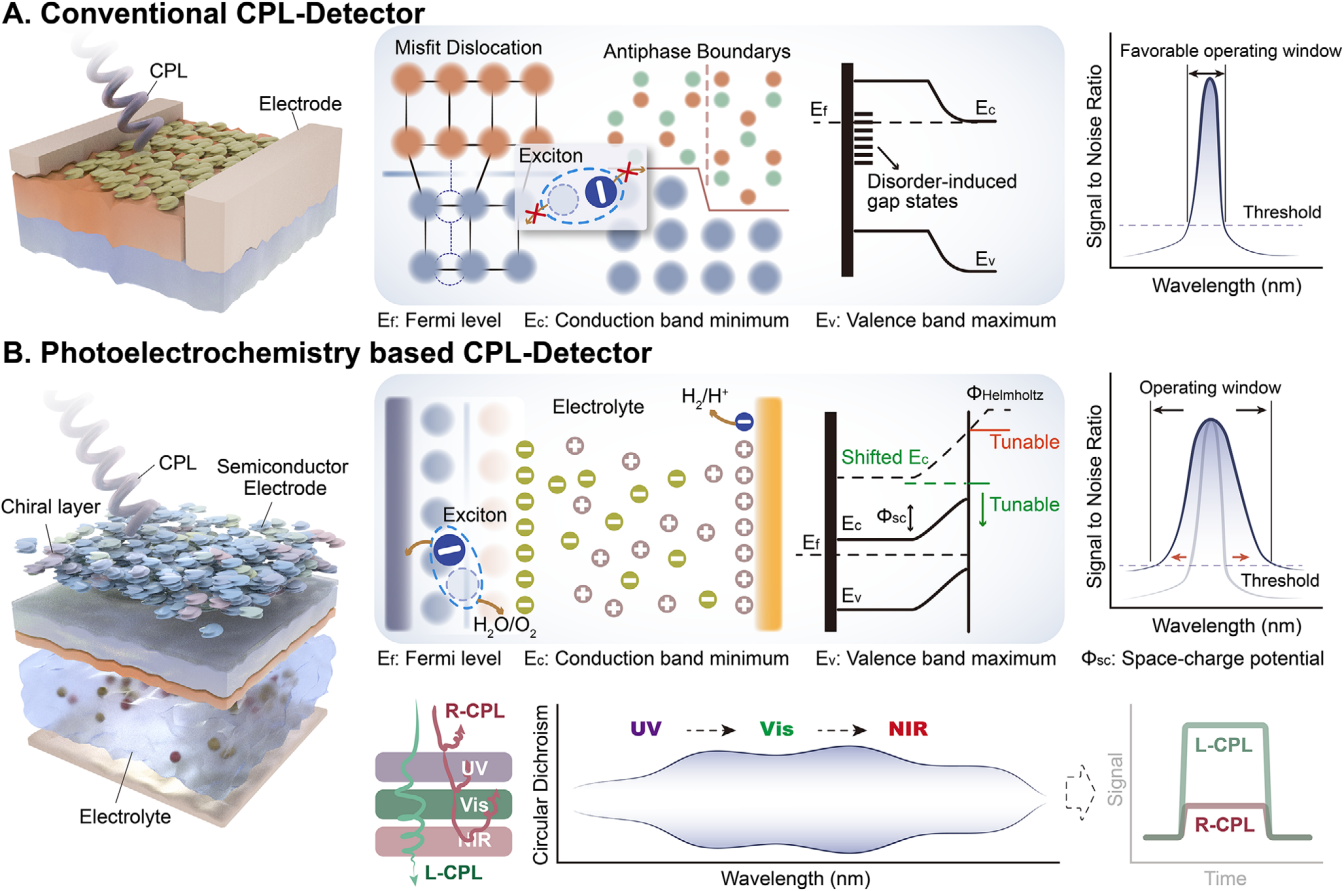
Conceptual comparison between conventional interface‐dependent solid‐state and photoelectrochemistry based CPL detectors. (A) Schematic illustration of the limitations of conventional CPL detectors. High‐fidelity readout is usually confined to a narrow favorable spectral window in solid‐state heterojunction‐based CPL detectors. CPL detectors, beyond which weak chirality‐dependent outputs are readily obscured by dark current and noise. At the defect‐prone solid‐solid junctions, structural imperfections such as misfit dislocations and antiphase boundaries can introduce disorder‐induced interfacial gap states, leading to Fermi‐level pinning and defect‐limited exciton dissociation and charge transport. (B) Interfacial features and optical modulation mechanism of the PEC‐type CPL detector. In the photoelectrochemical architecture, a broadband chiral modulation layer is coupled to a semiconductor/electrolyte transduction interface. The chiral layer provides chiral‐selective optical gating across the UV‐vis‐NIR region, while the dynamically regulated solid‐liquid interface alleviates pinning‐constrained charge transfer and enables broader‐window, higher‐fidelity CPL readout.

Herein, we develop a broadband photoelectrochemical‐type (PEC‐typed) CPL detection strategy by coupling a chiral modulation layer with a dynamically regulated photoelectrochemical transducer. Specifically, a modulation layer with multiple photonic bandgaps is constructed by layer‐by‐layer in‐situ polymerizing chiral polymers, whose optical response spans 360 to 830 nm, thereby enabling chirality‐selective gating of CPL across broad spectral windows. Meanwhile, a BiVO_4_‐RGO‐MXene photoanode is constructed as a dynamically regulated inorganic transducer through hierarchical assembly of BiVO_4_, RGO, and MXene components, which exhibits broadband photoelectrochemical response for electrical readout. By combining the modulation layer with photoanode, the chiroptical differences are subsequently converted into electrical signals by the photoelectrochemical transducer through adaptive interfacial charge regulation (Figure 1B). Distinct from conventional interface‐dependent solid‐state CPL detectors governed by static solid‐solid junctions (Figure 1A), this dynamically responsive PEC interface enables real‐time redistribution of interfacial charges, thereby mitigating trap‐assisted loss and noisy electrical readout, and improving the fidelity of weak chirality‐dependent photocurrent signals. In this way, a compact photodetector capable of converting broadband CPL inputs into electrical outputs within a single device is realized. Based on the chirality‐selective optical gating and adaptive interfacial charge transduction, a convolutional neural network (CNN) was employed to decode CPL‐encrypted images under opposite‐chirality noise interference, thereby enabling noise‐robust visual recognition within a compact chiroptical sensing platform. As a result, our strategy provides a new route toward high‐performance and compact CPL detection beyond the intrinsic constraints of fixed‐interface solid‐state architectures.

## 2 Result and Discussion

### 2.1 Dynamic Interfacial Charge Regulation in a Photoelectrochemical Transducer

**FIGURE 2:**
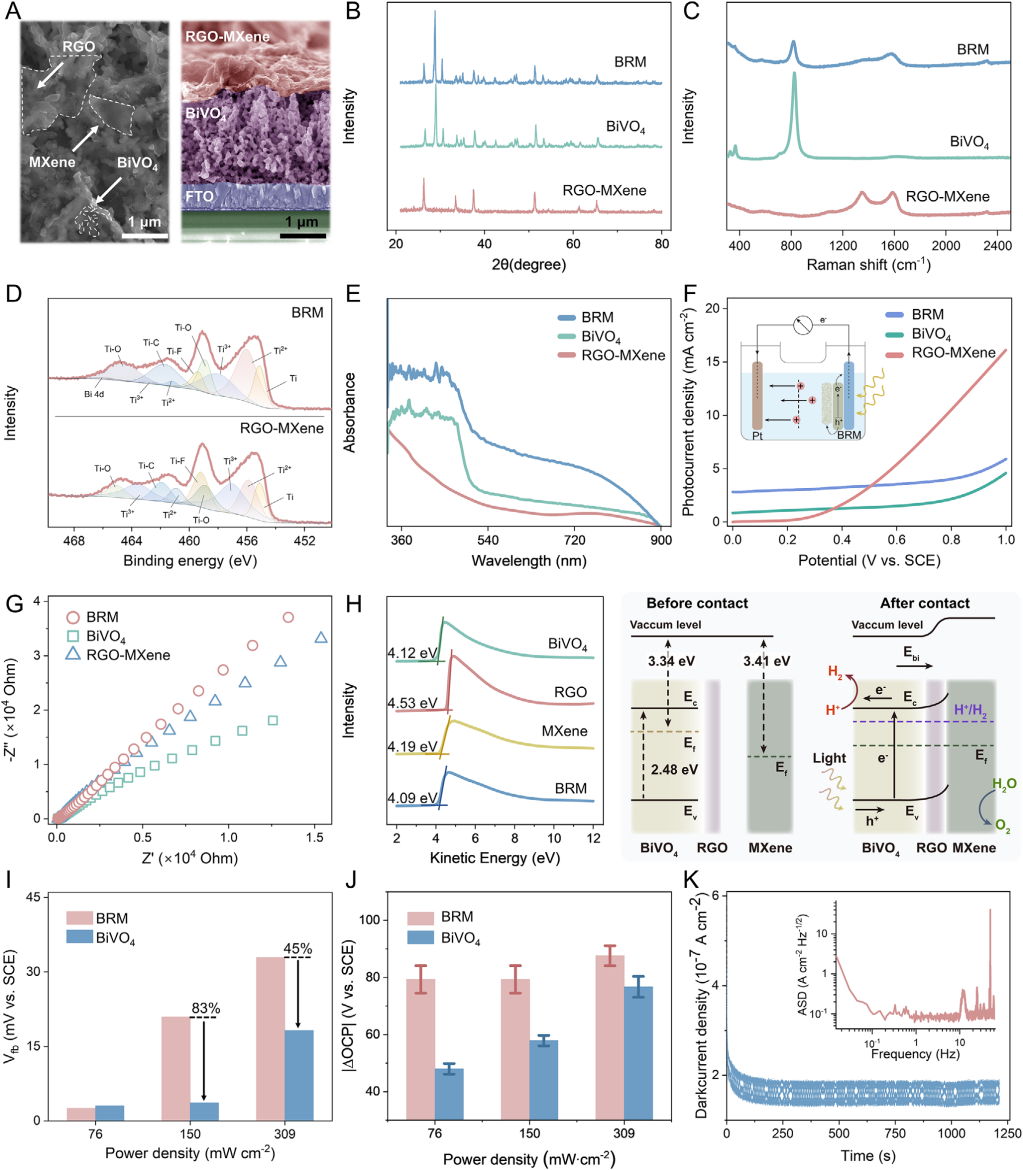
Characteristics of BRM photoelectrochemical transducer with dynamic interfacial charge regulation. (A) SEM and cross‐sectional images of the BRM. (B) XRD patterns, (C) Raman spectra of BRM, BiVO_4_, and RGO/MXene. (D) The XPS spectra of Ti 2p in BRM and RGO/MXene, indicating the interfacial chemical environment after assembly. (E) UV‐vis absorption spectra of BRM, BiVO_4_, and RGO/MXene. (F) The linear scanning voltammograms plots of BRM, BiVO_4_, and RGO/MXene; the inset shows the schematic illustration of the PEC charge‐transfer process under solar illumination (AM 1.5 G, 150 mW cm^−2^). (G) Nyquist plots of BRM, BiVO_4_, and RGO/MXene, revealing the reduced interfacial charge‐transfer resistance of BRM. (H) UPS spectra of BiVO_4_, RGO, MXene, and BRM. Schematic energy‐level alignment before and after contact, illustrating the built‐in interfacial charge‐transfer pathway in BRM. (I) The flat band potential (*V_fb_*), (J) Photo‐induced OCP shift (|ΔOCP|) of BRM and BiVO_4_ under 76, 150, 309 mW cm^−2^. (K) Dark‐current density time series at 0 V and frequency‐domain noise analysis (ASD) of BRM.

We adopt a photoelectrochemical interface‐engineering strategy to construct an inorganic photoanode featuring dynamically regulated interfacial charge behavior for high‐fidelity CPL detection. Specifically, a bottom‐up electrochemical deposition‐annealing route was employed to fabricate BiVO_4_, onto which RGO and MXene were subsequently integrated to form a ternary BiVO_4_‐RGO‐MXene (BRM) photoanode (Figure S1). SEM images reveal that BiVO_4_ grows into a worm‐like nanoporous network on the conductive substrate (Figure 2A), while film‐like RGO and few‐layer MXene nanosheets uniformly cover the framework, leading to a high‐surface‐area architecture (Figures S2 and S3). XRD patterns of BRM retain the characteristic diffraction peaks of the individual components, indicating that the crystalline phase identities of BiVO_4_ and RGO‐MXene are preserved after composite assembly (Figures 2B and S4). In contrast, Raman spectra display discernible band shifts and intensity redistribution in BRM relative to the pristine components, consistent with interfacial charge redistribution and strain coupling (Figures 2C and S5). XPS analysis further reveals pronounced changes in the chemical states of Ti after composite assembly, supporting charge redistribution at the composite interface (Figures 2D and S6). Moreover, BRM shows enhanced visible‐light absorption with a broadened absorption range toward longer wavelengths (Figure 2E). Correspondingly, Tauc analysis (Figure S7) gives an apparent optical bandgap of 2.39 eV for BRM. The weaker response beyond the BiVO_4_ band edge is discussed in terms of composite‐enabled sub‐bandgap excitation and interface‐assisted charge extraction. These results indicate effective interfacial coupling and a modified electronic environment in BRM, accompanied by broadened light absorption [33].

Electrochemical measurements were carried out in a standard three‐electrode photoelectrochemical configuration, where BRM served as the working electrode and a Pt electrode was used as the counter electrode. Linear sweep voltammetry (LSV) measurements reveal that BRM exhibits a photocurrent density of 2.82 mA cm^−2^ at 0 V vs SCE under illumination, indicating a pronounced self‐powered photocurrent with efficient carrier separation and extraction (Figure 2F). Electrochemical impedance spectroscopy shows a markedly contracted Nyquist semicircle for BRM under illumination, suggesting more efficient interfacial charge transfer at the BRM/electrolyte interface, which is favorable for the photoresponse (Figure 2G). Correspondingly, the Bode phase plot reveals a characteristic frequency shifted toward the low‐frequency region (Figure S8), indicating altered interfacial relaxation kinetics associated with charge accumulation and release at the BRM/electrolyte interfaces [34]. The band diagrams summarize the energy‐level alignment within the BRM architecture, constructed based on experimentally determined work functions (Figure 2H). Upon contact, multiple Schottky junctions are formed among BiVO_4_, RGO, and MXene, giving rise to built‐in electric fields that promote separation of photogenerated carriers, with electrons preferentially transported toward BiVO_4_ and holes toward MXene [35]. We further performed a quantitative assessment of interfacial pinning using frequency‐dependent Mott‐Schottky analysis and chopped‐light open‐circuit potential (OCP) measurements. Compared with bare BiVO_4_, the BRM electrode exhibits a markedly reduced flat‐band potential dispersion, ΔV_fb_, extracted at 3–5 kHz, with a more pronounced reduction under medium and high irradiance (by ≈83% and ≈45%) (Figures 2I and S9) [36, 37, 38]. Meanwhile, BRM consistently maintains a larger |ΔOCP| across all power densities (Figures 2J and S10). These results indicate that BRM effectively suppresses surface‐state charging and sustains a more substantial quasi‐Fermi level splitting, thereby alleviating interfacial Fermi‐level pinning. Such weakened pinning enables more direct and reliable optical‐to‐electrical transduction at the interface [39]. Such interfacial‐dominated kinetics are consistent with the observed self‐powered photoresponse, as dynamic charge accumulation and release at the solid‐liquid reduce reliance on defect‐sensitive, static charge transport constrained by solid‐solid junctions [40]. In addition, the light/dark OCP response yields a larger photovoltage for BRM, indicating enhanced interfacial band bending and a strengthened built‐in driving force that suppresses carrier recombination and promotes effective charge collection (Figure S11) [41]. Distinct from conventional solid‐solid heterojunctions, these Schottky interfaces operate in conjunction with a photoelectrochemical solid‐liquid junction, where interfacial ions dynamically participate in charge screening and redistribution under operation [42, 43]. This ion‐mediated interfacial regulation alleviates the impact of defect‐induced Fermi‐level pinning by enabling adaptive band bending and charge accommodation at the interface, rather than a rigid, lattice‐defined potential profile [44]. As a result, charge extraction in BRM is governed primarily by dynamically regulated solid‐liquid interfacial processes rather than defect‐limited transport across static solid‐solid junctions, which is consistent with the stable dark current observed at 0 V (Figure 2K) and the suppressed low‐frequency noise reflected in the amplitude spectral density (ASD; Figure 2K, inset) [45]. Electrolyte‐dependent control measurements further show that progressively replacing Na_2_SO_3_ with Na_2_SO_4_ decreases the photocurrent and light‐induced OCP shift, while increasing the interfacial impedance, apparent flat‐band potential, and dark‐current noise (Figure S12). Consequently, the dynamically regulated solid‐liquid interface endows BRM with a transduction architecture that is less sensitive to defect‐related interfacial trapping and pinning, thereby providing a low‐noise basis for efficient CPL signal conversion [46]. Electrolyte‐dependent control measurements were further performed to evaluate the contribution of the solid–liquid interface.

### 2.2 Chiral Multi‐Photonic‐Bandgap Film

**FIGURE 3:**
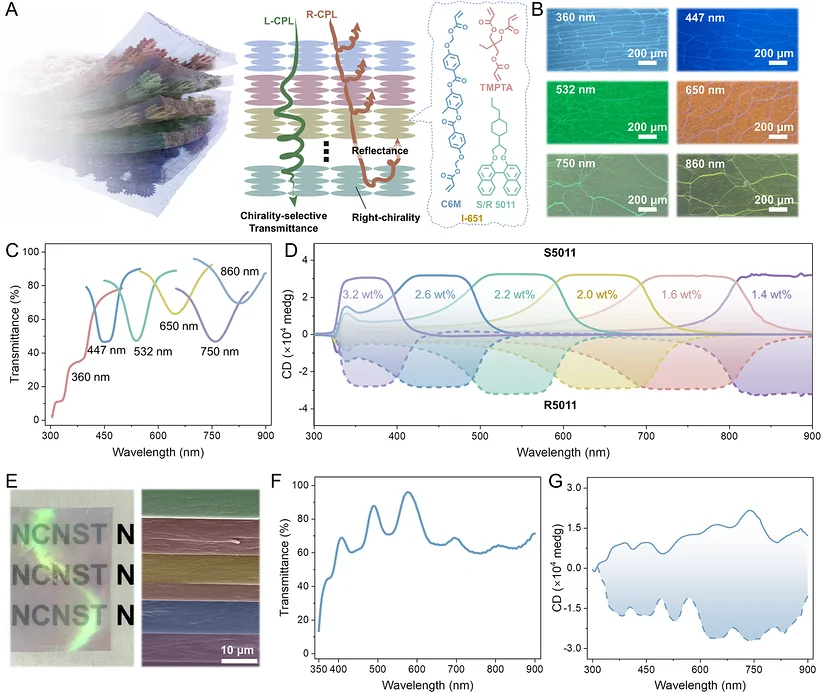
Multiphotonic‐bandgap composite CP films for broadband chiral filtering. (A) Schematic illustration of the L‐CPL‐transmissive multiphotonic bandgap composite CP films and the chemical composition of each layer. (B) Polarized optical microscopy (POM) images of single‐layer R‐CP films with different photonic bandgap positions. (C) Transmission spectra of single‐layer R‐CP films prepared with different concentrations of chiral dopant, showing tunable photonic bandgaps centered at 360, 447, 532, 650, 750, and 860 nm. (D) Corresponding CD spectra exhibiting mirror‐image signals at the designed photonic bandgap positions, indicating pronounced chiral discrimination. (E) Optical photograph and cross‐sectional morphology of the composite R‐CP film obtained via layer‐by‐layer in situ photopolymerization. (F) Transmission spectra of the R‐CP film, showing the coexistence of six sub‐reflection bands centered at 360, 447, 532, 650, 750, and 860 nm. (G) CD spectrum of the R‐CP film, demonstrating the preservation of chiral discrimination after multilayer integration.

To achieve chirality‐selective responses across UV‐vis‐NIR windows within a unified CPL detection platform, we constructed a chiral multi‐photonic‐bandgap film as a chiral modulation layer via a layer‐by‐layer in‐situ polymerizing strategy (Figure 3A). This chirality selectivity originates from the helical photonic crystal structure of chiral polymer (CP) films. Within the photonic bandgap, the film strongly reflects the circularly polarized component with the same chiral as the helix, while mainly transmitting the opposite‐chirality component, thereby converting the chirality information of CPL into a measurable difference in transmission intensity.

As a prerequisite for multilayer integration, several right‐handed chiral polymer (R‐CP) transmissive films with different photonic bandgap were fabricated by polymerizing monomer (C6M), chiral dopant (R‐5011), crosslinker (TMPTA), and photoinitiator (Irgacure 651). By tuning the concentration of the chiral dopant to regulate the helical pitch, six films with distinct bandgap positions spanning the target spectral range was prepared (Figure 3A). Polarized optical microscopy (POM) reveals that all six films exhibit characteristic oily‐streak textures (Figures 3B and S13). These textures are consistent with a predominantly planar cholesteric alignment, with the helical axis oriented approximately normal to the film plane (Figure S14). Correspondingly, the transmission spectra show that the reflection band can be continuously tuned by adjusting formulation, covering a spectral range from 360 to 830 nm (Figure 3C). In agreement with these photonic bandgap positions, mirror‐image circular dichroism (CD) signals appear at the corresponding wavelengths (Figure 3D), confirming the formation of well‐defined chiral photonic bandgaps.

Through layer‐by‐layer assembly, multiple reflection bands were integrated into a single freestanding film. Optical photographs clearly show that the stacked film remains good optical transparency, indicating that multilayer integration does not markedly compromise its visual transparency (Figure 3E). Cross‐sectional SEM images reveal distinct and continuous chiral layers with periodic ordering across the entire multilayer structure (Figure 3E). Transmission spectroscopy of the resulting chiral multi‐photonic‐bandgap film exhibits the coexistence of six sub‐reflection bands spinning 360 to 830 nm (Figures 3F and S15), confirming the successful integration of multiple photonic bandgaps within a single optical modulation layer. Corresponding mirror‐image CD signals are retained at each band (Figure 3G), indicating that the chiral discrimination capability is preserved after multilayer integration. Therefore, these results demonstrate that the stacked CP films function as an integrated chiral photonic layer that supports device miniaturization and provides a broadband chiral modulation platform for subsequent UV‐vis‐NIR CPL detection.

### 2.3 Circularly Polarized Light Photoelectrochemical Transducer

**FIGURE 4:**
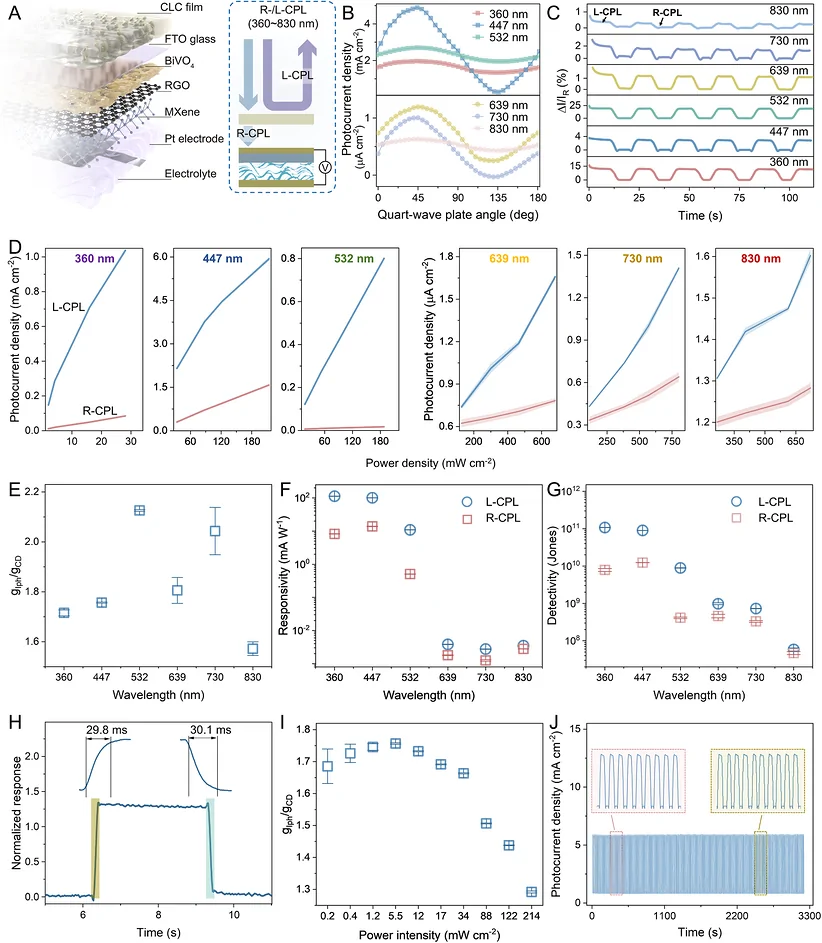
Feature of PEC‐type CPL photodetection. (A) Schematic illustration of PEC‐type CPL photodetector, constructed by coupling an S‐CP film with a BRM photodetector and its operation in a three‐electrode PEC system. (B) Photocurrent‐density dependence on quarter‐wave plate angle under illumination at 360, 447, 532, 639, 730, and 830 nm, showing periodic CPL discrimination across the full operating spectral window. (C) Time‐dependent normalized photocurrent response (Δ𝐼/𝐼_R_) under alternating L‐CPL and R‐CPL illumination at 360, 447, 532, 639, 730, and 830 nm. (D) Power‐density‐dependent photocurrent densities measured under L‐CPL and R‐CPL illumination at 360, 447, 532, 639, 730, and 830 nm, respectively. (E) Wavelength dependence of the ratio between the photocurrent dissymmetry factor and the CD dissymmetry factor (g_Iph_/g_CD_). (F) Wavelength‐dependent responsivity (R) of the device under L‐CPL and R‐CPL illumination. (G) Wavelength‐dependent specific detectivity (D*) at different wavelengths under L‐CPL and R‐CPL illumination. (H) Normalized transient photocurrent response of the R‐CP‐BRM detector recorded using an oscilloscope under TTL‐modulated 405 nm illumination. (I) Power‐density dependence of g_Iph_/g_CD_ at 447 nm. (J) Long‐term switching stability of the photocurrent response under repeated on/off illumination cycles; the insets show enlarged traces collected at different time intervals.

By coupling the right‐handed chiral multi‐photonic‐bandgap layer with the BRM transducer, a broadband L‐CPL‐discriminating photodetector was constructed. In this architecture, the chiral photonic layer selectively transmits L‐CPL and suppresses R‐CPL (Figures 4A and S16). The BRM layer, operating as a photoelectrochemical transducer in an electrolyte environment, enables self‐driven separation and collection of photogenerated carriers, thereby translating the optical asymmetry into a high‐ fidelity electrical signal (Figure S17).

Upon alternating L‐ and R‐CPL illumination, the device produces stable and reversible photocurrent switching from 360 to 830 nm, demonstrating a reliable broadband chirality‐selective response (Figure 4C). Moreover, the photocurrent exhibits a pronounced angular dependence on the ellipticity angle (Figures 4B and S18), confirming strong chiral selectivity and consistent polarization‐resolved photocurrent response across multiple spectral windows. In addition, the photocurrent increases monotonically with incident power density at all wavelengths while maintaining a clear difference between opposite circular states (Figure 4D; Figures S19 and S20). Notably, although the absolute photocurrent in the 639–830 nm range is about three orders of magnitude lower than that in the 360–532 nm range, the CPL‐dependent contrast remains well resolved. This result is consistent with the low‐noise and dynamically regulated interfacial charge‐transfer characteristics of the PEC transducer, which enable reliable electrical readout even when the optical response is substantially weakened. Accordingly, broadband and quantitative CPL discrimination can still be achieved within a single device architecture. To quantitatively assess the chiral discrimination capability, a chirality‐dependent photocurrent factor (g_Iph_/g_CD_) was extracted from time‐resolved photocurrent responses (Figure 4E). Consistently high g_Iph_/g_CD_ values of approximately 1.8 are maintained from 360 to 830 nm with only minor variation, confirming robust and broadband chiroptical sensitivity across the UV–vis‐NIR region compared to many previously reported works [8, 47, 48]. These results demonstrate that the PEC transduction pathway plays an important role in improving CPL detection performance by enabling broadband operation together with low‐noise, high‐fidelity electrical readout.

To further evaluate the chiral detection performance, the responsivity (R), defined as the ratio of photocurrent density to incident optical power, was extracted at representative wavelengths (Figure 4F). A clear responsivity contrast is observed between the two opposite circular polarization states across the entire spectral range from 360 to 830 nm. The responsivity reaches 110.8 mA W^−1^ at shorter wavelengths and remains appreciable at 3.1 mA W^−1^ at longer wavelengths, confirming broadband and wavelength‐consistent chirality discrimination. Furthermore, the specific detectivity (D*) was evaluated to assess weak‐signal discrimination under noise‐limited conditions (Figure 4G; Figures S21 and S22). Detectivity contrasts are consistently preserved across 360–830 nm, confirming excellent broadband chirality selectivity throughout the spectral window. Moreover, the device responds and recovers on the millisecond timescale, with rise and fall times of approximately 29.8 and 30.1 ms at 405 nm, respectively, as measured using an oscilloscope (Figure 4H). Meanwhile, the light/dark switching traces further show a stable baseline and highly reproducible photocurrent response with only small current fluctuations in both illuminated and dark states, providing time‐domain for the suppressed noise and stable interfacial charge transduction in BRM (Figure S23). The g_Iph_/g_CD_ value shows a clear power dependence but remains sufficiently high for effective chirality discrimination despite saturation at higher intensities (Figure 4I). Over 150 alternating L‐ /R‐CPL cycles, the device maintains stable and reversible photocurrent responses without obvious decay and with well‐preserved signal contrast, demonstrating excellent cycling stability and robust chirality discrimination (Figures 4J and S24). To assess the trade‐off between broadband spectral coverage and optical throughput, matched control measurements were performed using bare BRM and R‐CP‐BRM at 360, 639, and 830 nm (Figure S25). Although the six‐layer CP film reduced the optical throughput and consequently lowered the responsivity and specific detectivity, the close correspondence between their retention ratios indicates that no appreciable additional electrical‐noise penalty was introduced. More importantly, the helicity‐discrimination SNR increased from 0.255, 0.352, and 0.026 for bare BRM to 222.3, 14.7, and 7.99 for R‐CP‐BRM, respectively, demonstrating an acceptable trade‐off between absolute photodetection performance and broadband CPL‐discrimination capability. Consequently, the R‐CP‐BRM device provides a unified self‐powered platform for broadband CPL discrimination, combining quantitative chiral signal resolution with stable and reliable electrical output (Table S2).

### 2.4 CNN‐Assisted Noise‐Robust Recognition of Chirality‐Encrypted Images

**FIGURE 5:**
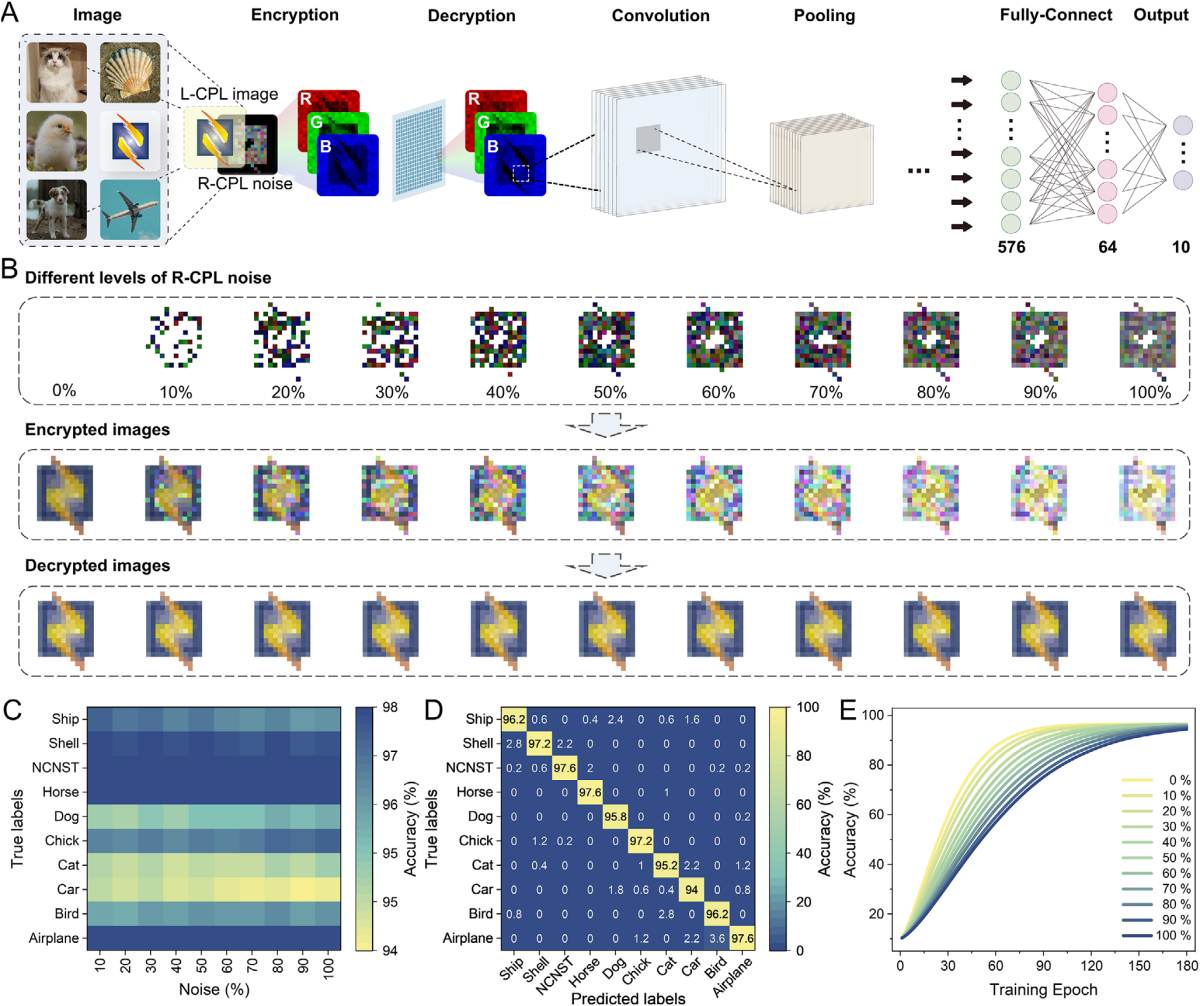
Noise‐robust CNN‐assisted recognition of CPL‐encrypted images. (A) Schematic illustration of the noise‐robust CPL‐encrypted image recognition process. The target image is encoded by L‐CPL, while background interference is encoded by R‐CPL noise. The R‐CP‐BRM device selectively transduces the L‐CPL component into photocurrent outputs and suppresses the opposite‐handed R‐CPL noise, generating decrypted electrical images for CNN architecture, including convolution, pooling, and fully connected layers. (B) Illustration of information encrypted hybrid images using different levels of R‐CPL noise and the corresponding decrypted images. (C) Recognition accuracy as a function of R‐CPL noise level. (D) Confusion matrix of CNN recognition under high R‐CPL noise. (E) Training accuracy under different R‐CPL noise levels of the NCNST image.

To further verify whether the broadband, low‐noise electrical outputs of the PEC‐typed CPL detector can be used for noise‐tolerant chiroptical information processing, we designed a CPL‐encrypted visual recognition task (Figure 5A). In this task, target visual information was encoded by L‐CPL, while background interference was encoded by opposite‐chirality R‐CPL noise, creating a chirality‐encrypted hybrid image. While conventional intensity‐based readouts suffer from substantially degraded visual contrast due to this noise, the R‐CP‐BRM detector selectively transduces the L‐CPL component into a pronounced photocurrent and suppresses the R‐CPL noise, achieving device‐level physical decryption. To demonstrate this, we performed a convolutional neural network (CNN)‐assisted recognition task using these CPL‐encrypted images. Decrypted photocurrent maps were generated from experimentally measured L‐CPL, R‐CPL, and dark‐current responses, which were subsequently fed into the CNN. We introduced different R‐CPL noise levels from 0% to 100% by assigning a controlled fraction of background pixels to the R‐CPL response distribution. As noise increased, target patterns became progressively obscured in the raw hybrid images. However, the decrypted electrical images from the R‐CP‐BRM readout retained recognizable target features across the entire noise spectrum, confirming efficient chirality‐selective optical gating (Figures 5B and S26).

Consequently, CNN recognition remained highly efficient, proving that the R‐CP‐BRM detector preserves sufficient discriminative features even under strong chiral interference. The accuracy map demonstrates consistent performance, with overall accuracy maintained at approximately 94% to 98% across all noise levels (Figure 5C). Confusion matrices under high noise exhibited dominant diagonal elements with minimal off‐diagonal misclassification (Figures 5D and S27). Furthermore, training curves converged continuously; although high R‐CPL noise initially slowed the learning process, the final accuracy reached a comparably high level (Figures 5E and S28). Unlike purely algorithmic denoising, this noise tolerance stems from device‐level chirality‐selective preprocessing, where undesired R‐CPL contributions are physically attenuated during optical‐to‐electrical conversion. This robustness aligns with the low‐noise charge‐transduction characteristics of the BRM photoelectrochemical interface. Its dynamically regulated solid‐liquid interface suppresses dark‐current fluctuations and low‐frequency noise, preserving weak chirality‐dependent features during image‐level readouts. Ultimately, the present PEC‐typed CPL detector not only enables broadband CPL discrimination but also serves as a robust physical preprocessing platform for noise‐tolerant chiroptical information decoding.

## 3 Conclusion

In summary, we have developed a broadband PEC strategy for CPL detection by coupling a chiral multi‐photonic‐bandgap modulation layer with a dynamically regulated BRM transducer. The stacked chiral polymer film converts the handedness of incident CPL into transmission‐intensity asymmetry across a broad spectral range, while the BRM photoanode translates this optical contrast into reliable electrical signals through self‐powered photoelectrochemical transduction. The dynamically regulated solid‐liquid interface of the BRM photoelectrochemical transducer effectively mitigates interfacial Fermi‐level pinning, suppresses low‐frequency noise, and stabilizes charge extraction, thereby enabling robust detection of subtle chirality‐dependent signals. As a proof of concept, the chirality‐selective photocurrent patterns are further exploited for CNN‐assisted recognition of CPL‐encrypted images, demonstrating the capability of the system to decode machine‐readable chiroptical information under opposite‐chirality noise interference. As a result, the compact device achieves high‐performance broadband CPL discrimination from 360 to 830 nm, delivering a high g_ph_, competitive responsivity and detectivity, millisecond response times, and excellent operational stability. This work presents a compact and general platform for converting broadband chiroptical information into electrical signals, offering a new design principle for high‐performance CPL optoelectronic devices.

## Author Contributions

P.D. conceived the idea and supervised the project. S.Z., H. J, P.D., and H.J. designed the experiments. S.Z. and H.J designed, synthesized, and characterized the materials. Y.Z. provided guidance on the CNN‐assisted recognition analysis. S.Z., H.J., and P.D. wrote and revised the manuscript. All authors analyzed and discussed the results and have given approval to the final version of the manuscript.

## Funding

The National Natural Science Foundation of China (22588301, 92256304); The National Key Basic R&D Research Program of Ministry of Science and Technology of the People's Republic of China (2021YFA1200303); the CHN Energy Investment Group GJNY‐26‐95(S930026046).

## Conflicts of Interest

The authors declare no conflicts of interest.

## Supporting information




**Supporting File**: advs77641‐sup‐0001‐SuppMat.docx.

## Data Availability

The data that support the findings of this study are available from the corresponding author upon reasonable request.
