# Supplementary material for: Dynamic Interfacial Charge Regulation for High‐Fidelity Broadband Circularly Polarized Light Detection
Source: Adv Sci (Weinh). 2026 Sep 11:e77641. Online ahead of print. doi: 10.1002/advs.77641 (PMC13564183; doi:10.1002/advs.77641)
Supplement: Supplementary file 1 [file ADVS-9999-e77641-s001.docx]

Supporting Information

Dynamic Interfacial Charge Regulation for High-Fidelity Broadband Circularly Polarized Light Detection

Siqin Zhou, Honghan Ji, Houchao Jing*, Xue Jin, Ying Zhao* and Pengfei Duan*

S. Zhou, H. Ji, H. Jing, X. Jin, P. Duan

Laboratory of Nanosystem and Hierarchical Fabrication, National Center for Nanoscience and Technology, Beijing 100190, P.R. China

E-mail: jinghc2025@nanoctr.cn; duanpf@nanoctr.cn

Y. Zhao

National Institute of Clean-and-Low-Carbon Energy, CHN Energy, Beijing, P.R. China

E-mail: ying.zhao.u@chnenergy.com.cn

S. Zhou, H. Ji, P. Duan

University of Chinese Academy of Sciences, Beijing, 101408, P.R. China

Keywords: circularly polarized light detection, photoelectrochemical transducers, chiral photonic films, dynamic interfacial charge regulation

S. Zhou and H. Ji contributed equally to this work.

**This file includes**

Experimental Section

Supplementary Methods

Supplementary Figures S1-28

Supplementary Table S1-2

References

**1. Experimental Section**

**Materials**

All the reagents were used as received without further purification. Bismuth Nitrate Pentahydrate (Bi(NO3)_3_·5H_2_O, 98%), Potassium iodide (KI, 99%), p-benzoquinone (BPQ, 99%), vanadyl acetylacetonate (VO(acac)_2_, 98%) were obtained from Aladdin. F-doped tin oxide glass (FTO) (1.5 cm × 2.3 cm) was purchased from South China Science & Technology Co., Ltd. Nitric Acid (70%, HNO_3_), ethanol, dimethyl sulfoxide (DMSO, 99%) were purchased from Macklin. Single layer graphene oxide dispersion (2 mg/mL, dispersion in H_2_O) was purchased from Meryer. Ti_3_C_2_T_x_ (MXene) few layer dispersion solution (5 mg/mL) was purchased from Compre Bonin Technology Co., Ltd. Polymer precursor 2-methyl-1,4-phenylene bis (4-((6-(acryloyloxy)-hexyl)oxy)benzoate) (C6M, 97%), cross-linking agent trimethylolpropane triacrylate (TMPTA, 95%), and photoinitiator 2,2-dimethoxy-2-phenylacetophenone (I-651, 99%) were purchased from Merck. The chiral dopant (S or R 5011, >98%) was provided by Jiangsu Hecheng Display Technology Co., Ltd. 360 nm (100 mW) laser, 447 nm (1 W) laser, 532 nm (1.5 W), 639 nm (1 W), 730 nm (1.5 W) and 830 nm (2 W) laser were purchased from Changchun New Industries Optoelectronics Technology Co., Ltd. λ/4 filter (350-850 nm) and linear polarizer (250 nm to 2.0 µm) were bought from Thorlabs.

**Characterizations**

Ultrahigh-resolution field-emission scanning electron microscope (FESEM, Hitachi-SU8220, Japan) was used to observe the morphologies of the materials using an accelerating voltage of 10 kV, and the elemental compositions were characterized by energy spectrometry. Powder X-ray diffraction analysis was carried out with a D/MAX-TTRIII (CBO) device, employing Cu K𝛼 radiation (𝜆 = 1.542 Å). The chemical properties of the layered materials were defined through Raman spectra (Renishaw InVia, 514 nm excitation laser). UV-vis transmittance spectra were recorded by UV-2600 equipped with an external diffuse reflectance accessory (DRA-2500). The X-ray photoelectron spectroscopy (XPS) spectra were characterized by Thermo Fisher ESCALAB250Xi, and the UPS spectra were measured using ESCALAB250Xi (Thermo Scientific, USA) instrument with a monochromatic He-I light source (21.22 eV). Transmittance spectra were measured using a Lambda 950 spectrophotometer. POM images were taken by a Leica DM2700M upright materials microscope. The performance of the PEC-type CPL photodetector was achieved on a CHI 760E potentiostat under different wavelengths (360, 447, 532, 639, 730, and 830 nm) lasers.

**Synthesis procedures**

**Fabrication of** **a BRM photoelectrochemical transducer with** **dynamic interfacial charge regulation:**

The precursor solution was prepared by dissolving 0.04 M Bi(NO_3_)_3_∙5H_2_O and 0.4 M KI in 50 mL H_2_O and then adding HNO_3_ to adjust the pH to 1.7. Then, the precursor solution was mixed with 20 mL of absolute ethanol (100%) containing 0.23 M benzoquinone and stirred vigorously for 5 min. A typical three-electrode cell was used for electrodeposition. The cathodic deposition was carried out at a constant potential of -0.1 V vs Ag/AgCl at room temperature for 5 min, and then BiOI electrodes were obtained. The dimethyl sulfoxide solution (DMSO) of 200 μL 0.2 M vanadyl acetylpyruvate (VO(acac)_2_) was dropped on the BiOI electrodes and heated by a muffle furnace in air at 450 °C for 2 h. Finally, there is excessive V_2_O_5_ on the annealed composite photoanode, so 1 M of NaOH is used to remove it. The resulting pure BiVO_4_ electrode was rinsed with deionized water. The electrodeposition experiments of RGO were performed by CV for 50 scan cycles from -1.5 V to 0.6 V in the nitrogen-purged 0.5 mg/mL precursor solutions with a scan rate of 50 mV/s, then we obtained the BiVO_4_/RGO. 100 μL of the MXene aqueous solution was dropped on the film of the BiVO_4_/RGO sample and dried in vacuum at 50 °C. Subsequently, an annealing step was used to enhance the interaction between FTO, BiVO_4_, RGO, and MXene. The BiVO_4_/RGO/MXene films were annealed for 30 min at 300 °C in an Ar atmosphere. Finally, the BRM (BiVO_4_/RGO/MXene) sample was obtained.

**Fabrication of single-layer chiral polymer film and multiphotonic bandgap stacked chiral polymer (CP) films:**

First, the formula for the single-layer chiral polymer film was prepared by dissolving C6M (1000 mg), TMPTA (62.4 mg), and I651 (20 mg) together with S/R-5011 in a toluene mixed solvent (20 mL). The amount of S/R-5011 was adjusted to tune the helical pitch and thus the photonic bandgap position. For representative photonic bandgap centers at 360, 447, 532, 639, 730, and 830 nm, the S/R-5011 masses were 32.0, 26.0, 22, 19.5, 15.5, and 13.5 mg, respectively. The solution was sonicated for 1 min to afford a homogeneous precursor. Then, a single-layer film (taking the 360 nm photonic bandgap formulation as an example) was fabricated by drop-casting 1 mL of the precursor solution onto a quartz substrate preheated to 100 °C. The solvent was allowed to evaporate until the mixture became viscous and exhibited LC character. A second quartz plate was then placed on top to promote uniform spreading of the LC mixture. After removing the assembly from the hot stage, the film was photopolymerized under 365 nm UV irradiation for 30 s to yield a solid cholesteric CP film. After that, multiphotonic bandgap stacked CP films were prepared through the in-situ polymerization process. To prevent organic-solvent-induced swelling or damage to the underlying CP layer during the deposition of subsequent layers, a 2 wt% aqueous poly(vinyl alcohol) (PVA) solution was spin-coated on the cured film as an interlayer protection layer and dried before the next coating step. The above casting-evaporation-lamination-UV curing cycle was repeated sequentially with precursor solutions having different photonic bandgap centers, producing a multilayer (e.g., six-layer) multiphotonic bandgap stacked CP film.

The central reflection wavelength of each cholesteric polymer layer is primarily determined by the average refractive index and helical pitch according to $\lambda_{c}=\bar{n}p$, while the corresponding reflection bandwidth is approximately described by $\Delta\lambda=\Delta np$. In the present system, the helical pitch was tuned by adjusting the concentration of the R- or S-5011 chiral dopant, thereby shifting the photonic bandgap to the desired spectral region. The film thickness mainly affects the reflection intensity, band-edge definition, and optical loss rather than directly determining the central wavelength.

Each chiral polymer layer was independently UV-cured before the subsequent layer was laminated, and a thin PVA layer was introduced between adjacent layers for transfer support and interfacial protection. Therefore, the final broadband optical response mainly originates from the superposition of the independently tuned photonic bandgaps. The internal interfaces may introduce additional Fresnel reflection, scattering, and weak interference effects. However, no pronounced band splitting, new resonance feature, or systematic displacement of the individual reflection bands was observed after multilayer integration, indicating that strong optical coupling between adjacent polymer layers is not dominant in the present stacked structure.

**Fabrication and measurement of L/R-CPL-discriminating photoelectrochemical circularly polarized light photodetector:**

On the BRM-free side of the FTO substrate, a thin PVA layer was first spin-coated as a transfer support. The pre-fabricated R/S-CP film was then aligned and laminated onto the PVA-coated surface from one edge. Trapped air was removed by softly pressing from the center outward using a compliant blade to ensure bubble-free, conformal contact over the active area. Finally, the laminated stack was briefly cured under mild heating and moderate pressure to strengthen interfacial adhesion, and the film edges were trimmed to define a clean optical window for subsequent measurements. The performance of the PEC-type CPL photodetector was used in a three-electrode system achieved on a CHI 760E potentiostat under different wavelengths (360, 447, 532, 639, 730, and 830 nm) of laser. The photoanode, counter electrode, and reference electrode were fabricated as photoelectrodes, a Pt sheet, and saturated calomel electrodes (SCE), respectively. The electrolyte consists of 1 M Na_2_SO_3_ and 0.5 M phosphate buffer solution (pH=7).

**Generation and switching of circularly polarized light:**

For the time-dependent photocurrent measurements, circularly polarized light was generated using a fixed linear polarizer followed by a broadband quarter-wave plate covering the investigated spectral range. The incident laser beam first passed through the linear polarizer to define a fixed linear polarization direction. The quarter-wave plate was mounted on a motorized rotational stage, with its fast axis positioned at the two opposite +45° and -45° orientations relative to the transmission axis of the linear polarizer to generate L-CPL and R-CPL, respectively, under the optical convention adopted in this work. During the photocurrent measurements, the motorized stage was alternated reproducibly between these two predefined angular positions, while the photocurrent was continuously recorded using the electrochemical workstation. For measurements at 360, 447, 532, 639, 730, and 830 nm, the corresponding laser source was selected while the linear polarizer-quarter-wave plate-device optical geometry was maintained unchanged. The resulting CPL beam was incident from the chiral polymer-film side of the R/S-CP-BRM device.

**CNN-assisted recognition of CPL-encrypted images**

An image-recognition model based on RGB photocurrent matrices was constructed to evaluate CPL-encrypted visual information processing. The photocurrent responses measured at 447, 532 and 639 nm were assigned to the blue, green and red channels, respectively. Ten image categories, including airplane, bird, car, cat, chick, dog, horse, NCNST, shell and ship, were converted into 20 × 20 three-channel matrices. The 20 × 20 sensing matrix was a device-informed virtual array constructed from experimentally measured photocurrent distributions rather than a physically fabricated detector array. Each pixel value in the RGB matrix was assigned according to the photocurrent response of the device at the corresponding wavelength. The L-CPL response was used to encode the target image information, while the R-CPL response was used as the opposite-handed background noise. Eleven R-CPL noise levels were introduced, including 0%, 10%, 20%, 30%, 40%, 50%, 60%, 70%, 80%, 90% and 100%.

For data construction, six groups of time-dependent photocurrent traces measured under L-CPL and R-CPL excitation at 447, 532 and 639 nm were used. Dark current, illuminated current and photocurrent response were extracted from each light on/off cycle according to

$$I_{\mathrm{ph}}=I_{\mathrm{light}}-I_{\mathrm{dark}}$$

Each raw current trace contained approximately 300 light on/off cycles. During extraction, the first and last 20% of each light or dark interval were removed to exclude transient switching processes, and the average value of the middle steady-state region was used as the current response of that cycle. The experimentally obtained photocurrent distributions were then randomly sampled to construct pixel responses in a virtual 20 × 20 device array. Therefore, the electrical image datasets were derived from experimentally measured device-response distributions rather than from direct image acquisition by a physical detector array.

To emulate the non-ideal output of a practical device array, several perturbation factors were introduced into the model, including pixel-to-pixel response variation, opposite-handed R-CPL noise leakage, readout noise, RGB channel crosstalk, neighbouring-pixel crosstalk and 8-bit analogue-to-digital conversion. The pixel-to-pixel response variation coefficient was set to 0.08, the opposite-handed R-CPL leakage coefficient to 0.25, the relative readout noise level to 0.035, the RGB channel crosstalk coefficient to 0.02, the neighbouring-pixel crosstalk coefficient to 0.04 and the ADC quantization depth to 8 bit.

For machine-learning analysis, independent RGB current matrices were generated at each R-CPL noise level and used for classification of the ten image categories. The CNN was trained for 180 epochs under each R-CPL noise level. For each category, 500 test samples were generated; therefore, each noise level contained 5000 test samples in total for statistical evaluation. Recognition accuracy was calculated as the ratio of correctly classified samples to the total number of test samples. Confusion matrices were constructed at each R-CPL noise level, where the diagonal elements represent correctly classified samples and the off-diagonal elements correspond to misclassified samples. Accordingly, the CNN demonstration represents a device-informed recognition task based on experimentally measured photocurrent distributions, rather than hardware imaging using a fabricated 20 × 20 detector array.

**2. Supplementary Methods**

**Optical bandgap from Tauc analysis**

The optical bandgap $E_{g}$ was estimated from Tauc plots using the relationship

$${(\alpha h\upsilon)}^{n}=B(h\upsilon-E_{g})$$

where $\alpha$ is the absorption coefficient, $h\upsilon$is the photon energy, $B$ is a proportionality constant, and the exponent 𝑛 depends on the transition type (typically 𝑛 = 1/2 for an indirect allowed transition and 𝑛 = 2 for a direct allowed transition). $E_{g}$ was obtained by extrapolating the linear region to the energy axis intercept.

**Work function from UPS cutoff**

The work function $\Phi$ was derived from ultraviolet photoelectron spectroscopy (UPS) using

$$\Phi=h\nu-\left| E_{F}-E_{\mathrm{cutoff}} \right|$$

where $h\nu$ = 21.22 eV, $E_{F}$ is the Fermi edge position on the UPS spectrum (referenced to the analyzer scale), and $E_{\mathrm{cutoff}}$ is the secondary-electron cutoff energy determined from the low-kinetic-energy onset.

**Photocurrent and photocurrent density**

The net photocurrent was calculated as

$$I_{\mathrm{ph}}=I_{\mathrm{light}}-I_{\mathrm{dark}}$$

where $I_{\mathrm{light}}$ is the measured current under illumination and $I_{\mathrm{dark}}$ is the current recorded in the dark under the same bias condition (here, 0 V for self-powered operation). The photocurrent density was obtained from

$$J_{\mathrm{ph}}=\frac{I_{\mathrm{ph}}}{S}$$

where $S$ is the effective illuminated area of the device.

**The flat-band potential (**$\mathbf{V}_{\mathbf{fb}}$**)**

The flat-band potential ($V_{\mathrm{fb}}$) was estimated from the Mott-Schottky plots. For an n-type semiconductor, the relationship between the space-charge capacitance and the applied potential is given by

$$\frac{1}{C_{\mathrm{SC}}^{2}}=\frac{2}{\varepsilon\varepsilon_{0}eN_{D}}(V-V_{\mathrm{fb}}-\frac{\mathrm{kT}}{e}）$$

where $C_{\mathrm{sc}}$ is the space-charge capacitance, $\varepsilon$ is the dielectric constant of the semiconductor, $\varepsilon_{0}$ is the vacuum permittivity, $e$ is the elementary charge, $N_{D}$ is the donor density, $V$ is the applied potential, $k$ is the Boltzmann constant, and $T$ is the absolute temperature. The linear region of the $\frac{1}{C_{\mathrm{SC}}^{2}}$ versus $V$ plot was linearly fitted and extrapolated to $\frac{1}{C_{\mathrm{SC}}^{2}}=0$. The corresponding x-intercept equals $E_{\mathrm{fb}}+\frac{\mathrm{kT}}{e}$, and was therefore taken as the flat-band potential because the $\frac{\mathrm{kT}}{e}$ term is negligible at room temperature. It should be noted that the extracted value represents an apparent flat-band potential and may be influenced by frequency dispersion and surface-state contributions.

Frequency-dependent Mott-Schottky measurements were performed at 0.5, 1, 3, and 5 kHz. The lower-frequency curves exhibited stronger dispersion because slow surface-state charging, trap-assisted processes, and electrochemical interfacial polarization could contribute to the measured capacitance. The apparent flat-band potentials obtained at 3 and 5 kHz were therefore selected for comparison because these curves exhibited more stable linear regions and reduced contributions from slow interfacial processes. The flat-band-potential dispersion was defined as $\Delta V_{\mathrm{fb}}=\left| V_{fb,3 kHz}-V_{fb,5 kHz} \right|$. In a depletion-capacitance-dominated interface, $\Delta V_{\mathrm{fb}}$ is nearly frequency-independent, whereas a larger $\Delta V_{\mathrm{fb}}$ reflects a stronger contribution from frequency-dependent surface-state or trap charging. Accordingly, the smaller $\Delta V_{\mathrm{fb}}$ of BRM indicates reduced interfacial-state-related capacitance dispersion and more stable apparent band energetics. Because the extracted values can also be affected by capacitance and series resistance, they are interpreted comparatively and together with the OCP and EIS results.

**Dark-current fluctuation analysis (time domain and frequency domain)**

To quantify the dark-current noise, a current-time trace 𝐼(𝑡) was recorded under complete darkness at 0 V. The sampling interval was $\Delta t$ (0.01 s in this work), and the total number of data points was 𝑁, giving a sampling frequency of $f_{s}=1/\Delta t$ and a total acquisition time of $T=N\Delta t$. To suppress the contribution from the DC component and slow baseline drift at low frequencies, the raw trace was first mean-removed to obtain $\delta I\left( t \right)=I\left( t \right)-\left\langle I \right\rangle$, when necessary, a linear detrending was additionally applied to 𝛿𝐼(𝑡) to minimize slow drift.

The current-noise power spectral density (PSD) was then estimated using the Welch method. Specifically, 𝛿𝐼(𝑡) was divided into overlapped segments of length 𝐿 (typically 50% overlap). Each segment was multiplied by a Hann window 𝑤[𝑛] to reduce spectral leakage, and a fast Fourier transform was applied to yield the frequency-domain amplitude 𝑋𝑘(𝑓) for the 𝑘-th segment. The single-sided current-noise PSD, 𝑆𝐼(𝑓), was obtained by averaging the spectral power over all segments and normalizing by the window energy and sampling frequency, yielding units of A Hz^−1^. With this normalization, the single-sided PSD is expressed as

$$S_{I}\left( f \right)=\frac{2}{f_{s}U}\left\langle\left| X_{k}(f) \right|^{2} \right\rangle$$

Where $U=\frac{1}{L}\sum_{n=0}^{L-1} \omega^{2}[n]$is the mean-square window factor, ⟨⋅⟩ denotes averaging over all segments, and the prefactor of 2 converts a two-sided spectrum into a single-sided spectrum (except at the DC and Nyquist components).

For clarity in comparison, the amplitude spectral density (ASD) was further reported as the square root of the PSD:

$$i_{n}\left( f \right)=\sqrt{S_{I}(f)}$$

where 𝑖𝑛(𝑓) has units of A Hz^−1/2^. When an area-normalized metric is required, the current can be converted to a current density$J\left( t \right)=I(t)/S$, where 𝑆 is the effective illuminated area (0.636 cm^2^ in this work). The same procedure then yields the corresponding density PSD 𝑆_𝐽_(𝑓) and ASD 𝑗_𝑛_(𝑓), with units of (A cm^−2^)^2^Hz^−1^ and A cm^−2^ Hz^−1/2^, respectively. ^1, 2^

**Responsivity**

The responsivity $R$ was evaluated by

$$R= \frac{I_{\mathrm{ph}}}{\mathrm{PS}}$$

where $P$ is the incident optical power density (in mW cm^−2^) measured using a calibrated power meter, and 𝑆 is the illuminated area.

**Specific detectivity**

The specific detectivity $D^{*}$ was calculated under the shot-noise-limited approximation using

$$D^{*}= \frac{R\sqrt{A_{D}}}{i_{\mathrm{shot}}^{2}}$$

where $A_{D}$ is the effective detection area, and $i_{\mathrm{shot}}^{2}$ is the current noise spectral density (in A Hz^−1/2^) at the frequency of interest. When the detector noise is assumed to be dominated by dark-current shot noise, $i_{\mathrm{shot}}^{2}$ can be approximated as

$$i_{\mathrm{shot}}^{2}= \sqrt{2qI_{\mathrm{DS}}}$$

which corresponds to a 1 Hz noise-equivalent bandwidth, where 𝑞 = 1.602◊10^−19^ C is the elementary charge and $I_{\mathrm{DS}}$ is the dark current at the same operating condition.

**Electrical dissymmetry factor for CPL discrimination**

The photocurrent dissymmetry factor was defined as

$$g_{I_{\mathrm{ph}}}= \frac{2\left| I_{L} - I_{R} \right|}{I_{L} + I_{R}}$$

where $I_{L}$ and $I_{R}$ denote the photocurrent densities measured under L- and R-CPL, respectively, at identical wavelength and incident power density. This quantity describes the electrical discrimination of CPL helicity.

**Optical dissymmetry factor from CD and absorbance**

The CD signal (in mdeg) was converted to differential absorbance via

$$\mathrm{CD}\left[ \mathrm{mdeg} \right]=32980 \times\Delta A$$

where $\Delta A=A_{L}-A_{R}$ is the absorbance difference for L- versus R-CPL. The corresponding optical dissymmetry factor was then defined as

$$g_{\mathrm{CD}}= \frac{\mathrm{CD}\left[ \mathrm{mdeg} \right]}{32980 \times A}= \frac{\Delta A}{A}$$

where 𝐴 is the absorbance at the same wavelength obtained from UV-vis measurements. This normalization allows direct comparison of intrinsic chiroptical asymmetry among samples with different optical densities.

To evaluate how efficiently the device converts an optical helicity asymmetry into an electrical output contrast, we introduce the ratio $g_{I_{\mathrm{ph}}}/g_{\mathrm{CD}}$ as a normalized figure-of-merit.

This metric is conceptually useful for two reasons. First, it decouples electrical discrimination from the absolute magnitude of CD that can vary with film thickness, optical density, and spectral overlap, thereby enabling more meaningful comparisons across wavelengths and samples. Second, in cascaded architectures—where a chiral optical element (e.g., a photonic film) modulates the transmitted photon flux and a downstream transducer converts the flux difference into current. $g_{I_{\mathrm{ph}}}/g_{\mathrm{CD}}$ provides a compact measure of the effective transduction gain from optical asymmetry to electrical contrast. Values of$g_{I_{\mathrm{ph}}}/g_{\mathrm{CD}}$>1 indicate that the electrical readout exhibits a larger relative contrast than expected from the intrinsic optical dissymmetry alone, consistent with additional amplification arising from device-level transport, interfacial separation, and collection processes. Accordingly, $g_{I_{\mathrm{ph}}}/g_{\mathrm{CD}}$ serves as a practical benchmark for assessing and optimizing the conversion efficiency in CPL-sensitive photoelectrochemical detection.

**Quantification of optical-throughput retention and CPL-discrimination performance**

To evaluate the optical-throughput penalty introduced by the six-layer chiral polymer film, the incident optical power and transmitted optical power were measured under L- and R-CPL illumination. For each helicity $h$, where $h$ = L or R, the transmittance of the blank FTO substrate was calculated as

$$T_{FTO,h}=\frac{P_{FTO,h}}{P_{0,h}}$$

where $P_{0,h}$ is the incident optical power measured at the sample position and $P_{FTO,h}$ is the optical power transmitted through the blank FTO substrate.

The transmittance of the six-layer CP/FTO structure was calculated as

$$T_{CP/FTO,h}=\frac{P_{CP/FTO,h}}{P_{0,h}}$$

where $P_{CP/FTO,h}$ is the optical power transmitted through the six-layer CP/FTO structure.

The helicity-dependent optical-throughput retention introduced by the CP film was then defined as

$$\eta_{T,h}=\frac{T_{CP/FTO,h}}{T_{FTO,h}}\times100\%=\frac{P_{CP/FTO,h}}{P_{FTO,h}}\times100\%$$

The average optical-throughput retention was calculated as

$$\eta_{T,avg}=\frac{\eta_{T,L}+\eta_{T,R}}{2}$$

The net photocurrent under each polarization state was calculated from

$$I_{ph,h}=\left| I_{light,h}-I_{\mathrm{dark}} \right|$$

and the responsivity was calculated as

$$R_{h}= \frac{I_{ph,h}}{P_{0,h}}$$

where $P_{0,h}$ is the optical power incident on the front surface of the complete device. When current density and optical power density were used, the equivalent expression $R_{h}=J_{ph,h}/p_{0,h}$ was applied.

The average responsivity of each device was calculated as

$$R_{T,avg}=\frac{R_{L}+R_{R}}{2}$$

and the responsivity retention of the integrated device relative to bare BRM was defined as

$$\eta_{R}=\frac{R_{avg,R-CP-BRM}}{R_{avg,BRM}}\times100\%$$

The specific detectivity was calculated using

$$D^{*}= \frac{R\sqrt{A_{D}}}{i_{\mathrm{shot}}^{2}}$$

The average specific detectivity was calculated from the L- and R-CPL values, and the specific detectivity retention was defined as

$$\eta_{D^{*}}=\frac{D_{avg,R-CP-BRM}^{*}}{D_{avg,BRM}^{*}}\times100\%$$

To quantify the ability of the device to distinguish opposite CPL helicities, repeated L- and R-CPL steady-state responses were analyzed. The helicity-discrimination signal-to-noise ratio was defined as

$$\mathrm{SNR}_{\mathrm{CPL}}=\frac{\left| \mu_{L}-\mu_{R} \right|}{\sqrt{\sigma_{L}^{2}+\sigma_{R}^{2}}}$$

where $\mu_{L}$ and $\mu_{R}$ are the mean steady-state responses obtained from repeated L- and R-CPL cycles, respectively, and $\sigma_{L}$ and $\sigma_{R}$ are the corresponding cycle-to-cycle standard deviations. When the incident powers of L- and R-CPL were not identical, responsivity rather than raw photocurrent was used in the SNR calculation.”

**3. Supplementary Figures**

Figure S1. The preparation process of the BRM photoelectrochemical transducer.

Figure S2. (A) SEM images of BRM. (B) the elemental ratios of BRM. (C-H) EDS of C, O, Ti, V, Sn and Bi elements. This result indicates the successful preparation of BRM.

Figure S3. SEM images of (A) RGO; (B) MXene.

Figure S4. XRD patterns of (A) RGO; (B) MXene; (C) FTO.

(A) RGO shows no distinct graphitic (002) reflection, consistent with a largely film-like structure, whereas (B) MXene exhibits the characteristic low-angle (002) peak; the reflections in (C) originate from the FTO substrate and serve as a baseline for separating substrate contributions from the BRM XRD pattern.

Figure S5. Raman spectra of (A) RGO; (B) MXene.

(A) Raman spectrum of RGO, exhibiting the characteristic D (≈1350 cm^-1^) and G (≈1580 cm⁻^1^) bands that reflect a defect-containing sp² carbon network. (B) Raman spectrum of MXene, showing typical lattice/termination-related vibrational features at low Raman shifts, consistent with MXene-type frameworks.

Figure S6. The XPS spectra of (A) Bi 4f in BRM and BiVO_4_; (B) V 2p in BRM and BiVO_4_; (C) C 1s in BRM, RGO/MXene, RGO, and MXene.

Compared with pristine BiVO_4_, the Bi 4f and V 2p core levels in BRM exhibit a discernible binding-energy shift, which is consistent with interfacial charge redistribution induced by coupling BiVO_4_ with the conductive RGO/MXene framework. The C 1s envelopes further verify that the BRM composite simultaneously retains the MXene-related C-Ti component and the RGO-related carbon/oxygen functionalities, supporting the effective integration of the ternary components without introducing additional carbon-phase impurities.

In the BRM architecture, BiVO_4_ primarily serves as the semiconductor absorber and photocarrier-generation component. RGO forms a conductive bridging network that facilitates carrier transport and separation. MXene provides a highly conductive two-dimensional interface that promotes interfacial charge redistribution and electronic-ionic coupling at the solid-liquid interface^3, 4, 5, 6^. BiVO_4_ was selected as the basis of the PEC transducer because its relatively narrow bandgap among commonly used oxide photoelectrodes provides substantial visible-light photoresponse, making it more suitable than typical wide-bandgap semiconductors for evaluating broadband CPL readout. The incorporation of RGO and MXene further improves the charge-extraction and interfacial characteristics of this BiVO_4_-based architecture. Importantly, BRM itself is achiral and serves only as the photoelectrochemical transducer, while CPL selectivity is independently introduced by the stacked chiral photonic polymer film. Previous studies have demonstrated BiVO_4_/RGO and BiVO_4_/MXene architectures for enhanced photoelectrochemical conversion and self-powered photodetection, respectively. However, to the best of our knowledge, the exact BiVO_4_-RGO-MXene ternary architecture has not previously been employed for direct CPL detection.

Figure S7. Tauc plots of BRM and BiVO_4_.

The apparent optical bandgap of BRM is approximately 2.39 eV, corresponding to an absorption edge of approximately 519 nm. Therefore, the response at 650-830 nm cannot originate from conventional band-to-band excitation of BiVO_4_. Previous studies have demonstrated that sub-bandgap states in BiVO_4_ can contribute to measurable photocurrent and that oxygen-vacancy-associated states influence carrier trapping, detrapping, and extraction^7, 8^. In BRM, the broadband optical background and conductive pathways provided by RGO/MXene, together with electronic coupling at the composite interfaces, may facilitate the conversion and extraction of carriers generated through these weak sub-bandgap pathways^6^. Accordingly, the long-wavelength response is considered to involve the combined effects of BiVO_4_ sub-bandgap states and interface-assisted charge extraction. Because the relevant intermediate states were not spectroscopically resolved, no single microscopic transition is assigned as the exclusive origin.

Figure S8. The Bode plots of BRM, BiVO_4_, and RGO/MXene. The characteristic frequency at the phase maximum (f_max_) is highlighted for each sample (BRM: 0.12 Hz; BiVO₄: 0.38 Hz; RGO/MXene: 8.25 Hz).

Figure S9. Mott-Schottky curves of BRM and BiVO_4_ at different frequencies (the colors from light to dark represent 0.5, 1, 3, and 5 kHz, respectively), under (A) 76, (B) 150, (C) 309 mW cm^-2^.

At irradiance levels of 76, 150, and 309 mW cm^-2^, pristine BiVO_4_ exhibits pronounced frequency dispersion and stronger low-frequency non-ideal behavior, indicating substantial contributions from surface-state and trap charging. In comparison, BRM shows reduced dispersion across the measured frequency range. The apparent flat-band-potential dispersion, defined as $\Delta V_{\mathrm{fb}}=\left| V_{fb,3 kHz}-V_{fb,5 kHz} \right|$, is consistently smaller for BRM, with reductions of approximately 83% and 45% under 150 and 309 mW cm^-2^, respectively. These results indicate that the BRM interface is less affected by frequency-dependent interfacial charging, consistent with improved carrier extraction and alleviated surface-state-related pinning.

Figure S10. OCP curves of BRM and BiVO_4_ under different optical powers (the colors range from light to dark as 76, 150, 309 mW cm^-2^).

At the BRM/electrolyte junction, the semiconductor space-charge region is coupled to an electrical double layer containing mobile ions and redox species. Changes in the surface photocarrier density under illumination induce reversible ionic redistribution and modify the interfacial potential distribution and band bending^9, 10, 11^. In the sulfite-containing electrolyte, interfacial hole transfer additionally limits prolonged surface-hole accumulation. Coupled with the conductive RGO/MXene transport network, this ionic and redox compensation reduces slow surface-state charging and facilitates reversible charge accumulation and release. This interpretation is consistent with the smaller charge-transfer resistance, reduced Mott-Schottky frequency dispersion, larger light-induced OCP shift, and suppressed dark-current fluctuations of BRM. Consequently, the stabilized photocurrent baseline helps preserve the relatively small L-CPL/R-CPL current difference during repeated measurements. Here, adaptive charge redistribution refers to the reversible response of the electrical double layer and interfacial redox environment to changes in photocarrier density.

Figure S11. OCP plots in dark and under illumination of BiVO_4_, RGO, MXene, and BRM.

Figure S12. Electrolyte-dependent interfacial charge regulation of the R-CP-BRM device under 447 nm illumination. (A) Photocurrent-density-potential curves. (B) Nyquist plots under illumination. (C) Chopped-light OCP responses and (D) corresponding ∣ΔOCP∣. (E) Dark-current-density traces and (F) current-density ASD spectra. (G-I) Frequency-dependent Mott-Schottky plots in 1.0 M Na_2_SO_3_, 0.5 M Na_2_SO_3_ + 0.5 M Na_2_SO_4_, and 1.0 M Na_2_SO_4_, respectively. (J) High-frequency apparent flat-band potentials averaged from the values extracted at 3 and 5 kHz.

To evaluate the contribution of the electrolyte environment to charge extraction and signal fidelity, control measurements were performed under identical 447 nm illumination using 1.0 M Na_2_SO_3_, 0.5 M Na_2_SO_3_ + 0.5 M Na_2_SO_4_, and 1.0 M Na_2_SO_4_ electrolytes. With decreasing Na_2_SO_3_ concentration, both the overall photocurrent and the absolute L/R-CPL current difference progressively decreased, while the helicity-selective response direction remained unchanged. The enlarged Nyquist semicircles and the decrease in the light-induced OCP shift from approximately 47 mV to 28 and 26 mV indicate less efficient interfacial charge transfer after replacing Na_2_SO_3_ with Na_2_SO_4_.

The high-frequency apparent flat-band potential, averaged from the values extracted at 3 and 5 kHz, shifted positively from 0.074 V in 1.0 M Na_2_SO_3_ to 0.124 V in the mixed electrolyte and 0.172 V vs SCE in 1.0 M Na_2_SO_4_. Because the measurements were performed under illumination, these values are regarded as apparent flat-band potentials and may contain contributions from surface states, ionic adsorption, and Helmholtz-layer capacitance. In addition, the integrated RMS dark-current-density noise over 0.1-5 Hz increased from 3.52×10^-9^ to 3.99×10^-9^ and 8.59×10^-9^ A cm^-2^, respectively. These results demonstrate that the chiral polymer film determines the CPL selectivity, whereas electrolyte-mediated ionic and redox processes make an important contribution to carrier extraction and weak-signal preservation.

Figure S13. (A-F) Polarized optical microscopy (POM) images for single-layer S-CP films prepared with different chiral dopant ratios.

Figure S14. Higher-magnification cross-sectional SEM images of the chiral photonic polymer films. (A) Film/substrate interface and (B) film/film interface between adjacent chiral polymer layers. The periodic lamellar textures observed approximately parallel to the corresponding interfaces support a predominantly planar cholesteric alignment with the helical axis mainly oriented along the film-normal direction.

Higher-magnification cross-sectional SEM images reveal periodic lamellar textures at both the film/substrate interface and the interface between adjacent chiral polymer layers. The textures are arranged approximately parallel to the corresponding interfaces, supporting a predominantly planar cholesteric alignment with the helical axis mainly oriented along the film-normal direction. Because SEM does not directly resolve individual molecular orientations, these observations are interpreted as morphological evidence for the preferred helical organization.

Figure S15. Transmission spectra of the single-layer S-CP films.

Figure S16. Photographs of the integrated chiral photonic-photoelectrochemical CPL detector. (A) Front view from the chiral multiphotonic-bandgap film side. (B) Side view showing the laminated integration of the chiral photonic layer with the photoelectrochemical transducer. (C) Rear view from the FTO-supported BRM photoelectrode side.

Figure S17. Experimental configuration for CPL-dependent photoelectrochemical measurements. The selected laser beam was sequentially passed through a fixed linear polarizer and a broadband quarter-wave plate mounted on a motorized rotational stage. L-CPL and R-CPL were generated by positioning the fast axis of the quarter-wave plate at the two opposite +45° and -45° orientations relative to the transmission axis of the linear polarizer. During the time-dependent photocurrent measurements, the motorized stage reproducibly alternated between these two predefined positions, while the photocurrent of the R/S-CP-BRM working electrode was continuously recorded in a three-electrode photoelectrochemical configuration. WE, working electrode; RE, reference electrode; CE, counter electrode.

Figure S18. Polar-coordinate plots of photocurrent response versus quarter-wave plate (QWP) angle for devices with opposite chirality (R-CP-BRM and S-CP-BRM) under 447 nm excitation.

Under the same excitation conditions, two devices using opposite-handed chiral films (R-CP-BRM and S-CP-BRM) show opposite angular dependences of the photocurrent, directly confirming that the chiral filtering layer determines the selective direction of the device for L/R-CPL.

Table S1. Wavelength and power density of the incident light in PEC-type CPL detection.

|  | Ⅰ (mW cm^-2^) | Ⅱ (mW cm^-2^) | Ⅲ (mW cm^-2^) | Ⅳ (mW cm^-2^) |
| --- | --- | --- | --- | --- |
| 360 nm | 2.05 | 4.22 | 16.02 | 28.11 |
| 447 nm | 33.6 | 87.5 | 121.9 | 214.1 |
| 532 nm | 17.5 | 52.5 | 89.1 | 188.1 |
| 639 nm | 127.7 | 301.0 | 465.0 | 681.0 |
| 730 nm | 115.1 | 383.0 | 569.0 | 803.0 |
| 830 nm | 267.1 | 405.0 | 613.0 | 722.0 |

Figure S19. The I-t curves of the R-CP-BRM CPL detector to switching between L- and R-CPL under (A) 360, (B) 447, (C) 532, (D) 639, (E) 730, (F) 830 nm, and different power densities.

Across the UV-vis-NIR range, the R-CP-BRM photodetector exhibits repeatable L/R-CPL switching with clear power-dependent photocurrent amplitudes, confirming robust self-powered photoelectrochemical transduction under different excitation conditions.

Figure S20. The I-t curves of the S-CP-BRM CPL detector to switching between L- and R-CPL under (A) 360, (B) 447, (C) 532, (D) 639, (E) 730, (F) 830 nm, and different power densities.

The response characteristics of L/R-CPL are reversed compared with those in Figure S20. These results confirm that the PEC readout remains robust for both opposite circular polarization states over 360-830 nm, providing a consistent basis for evaluating polarization-dependent responses and the derived figures of merit.

Figure S21. The $R$ and $D^{*}$of the R-CP-BRM CPL detector to switching between L- and R-CPL under (A) 360, (B) 447, (C) 532, (D) 639, (E) 730, (F) 830 nm, and different power densities.

Across all wavelengths, both $R$ and $D^{*}$ show a decreasing trend with increasing optical power density, but the chirality-dependent separation between the L-CPL and R-CPL traces remains clear over the full UV-vis-NIR window.

Figure S22. The $R$ and $D^{*}$of the S-CP-BRM CPL detector to switching between L- and R-CPL under (a) 360, (b) 447, (c) 532, (d) 639, (e) 730, (f) 830 nm, and different power densities.

The response characteristics of L/R-CPL are reversed relative to those in Figure S22.

Figure S23. Transient photoresponse at (a) 360, (b) 447, (c) 532, (d) 639, (e) 730, (f) 830 nm showing the $t_{r}$ and $t_{f}$ under L-CPL and R-CPL.

The wavelength-dependent transient photocurrent curves were recorded using the electrochemical workstation and are presented to demonstrate stable and repeatable photoresponse at different wavelengths. Because the acquisition bandwidth, sampling interval, and internal filtering differ from those of the oscilloscope measurement, these traces are not used for quantitative response-time comparison.

Figure S24. Long-term self-powered stability of R-CP-BRM at 0 V under (A) 360, (B) 447, (C) 532, (D) 639, (E) 730, (F) 830 nm illumination (>150 alternating L-CPL and R-CPL illumination cycles).

Under identical PEC measurement conditions, the device maintains stable, highly reproducible photocurrent switching over >150 illumination cycles across the UV-vis-NIR range, with no abrupt degradation during continuous operation. This wavelength-resolved cycling robustness supports the reliability of the chiral-filter/PEC-transduction cascade for broadband CPL readout.

Figure S25. (A) Wavelength-dependent L-CPL, R-CPL, and average optical-throughput retention of the six-layer R-CP/FTO structure. (B) Responsivity and specific detectivity retention of the integrated R-CP-BRM device relative to bare BRM at representative wavelengths of 360, 639, and 830 nm. (C) Helicity-discrimination signal-to-noise ratios of bare BRM and R-CP-BRM.

Figure S26. CPL-encrypted images under different R-CPL noise levels.

CPL-encrypted images constructed under different R-CPL noise levels from 0% to 100%, together with their corresponding R, G and B channel maps. The gradual degradation of image contrast with increasing R-CPL noise illustrates the interference effect of opposite-handed CPL noise before R-CP-BRM decryption and CNN recognition.

Figure S27. Confusion matrices of CNN recognition under (A) 0%, (B) 10%, (C) 20%, (D) 30%, (E) 40%, (F) 50%, (G) 60%, (H) 70%, (I) 80%, (J) 90% R-CPL noise levels.

Confusion matrices of CNN recognition results obtained under R-CPL noise levels from 0% to 90%. The dominant diagonal elements in all matrices indicate that the decrypted photocurrent images can be reliably classified across different image categories. Only minor off-diagonal misclassification is observed as the R-CPL noise level increases, confirming the noise-robust recognition capability enabled by chirality-selective R-CP-BRM readout.

Figure S28. CNN training accuracy of (A) airplane, (B) bird, (C) car, (D) cat, (E) chick, (F) dog, (G) horse, (H) shell, (I) ship under different R-CPL noise levels.

Category-dependent training accuracy curves of the CNN under different R-CPL noise levels. Although increasing R-CPL noise slows the early-stage convergence, all categories finally reach high recognition accuracy after sufficient training epochs, confirming the noise-robust feature retention in the decrypted electrical images.

| **Table S2. Comparison of representative CPL photodetectors based on different chiral layers and signal-transduction architectures.** | | | | | | | | | | |
| --- | --- | --- | --- | --- | --- | --- | --- | --- | --- | --- |
|  | **Device sarchitecture** | **CPL discrimination mechanism** | **Spectral mode** | **Bias and electrical-readout strategy** | **Key architectural feature** | **Responsivity, R (A W^-1^)** | **Detectivity, D* (Jones)** | **Response time/ bandwidth** | **Electrical dissymmetry factor** | **Ref.** |
| Grey et al., Adv. Funct. Mater. 2019 | Chiral nematic cellulose nanocrystal solid electrolyte | Selective LCPL reflection/ RCPL transmission followed by the a-IGZO photoresponse | 405 nm | V_GS_ = -2 V | Photonic solid-electrolyte-gated FET; no dynamic semiconductor/electrolyte interfacial regulation. | — | — | — | I_ph,RCPL_ ≈ 2 × I_ph,LCPL_ | [12] |
| Chen et al., Nat. Commun. 2019 | (R/S-α-PEA)PbI_3_ chiral hybrid perovskite | Differential CPL absorption in an intrinsically chiral semiconductor | 365, 395, 430, and 530 nm | 20 V | Single-layer chiral photoconductor; static solid-state contacts. | 0.797 | 7.1 × 10^11^ | 150 Hz (-3 dB) | g_res_ = 0.10 at 395 nm | [13] |
| Zhang et al., Nat. Commun. 2021 | π-Extended PDI double-[7]heterohelicene | Intrinsic molecular CD combined with phototransistor gain | Broadband UV-NIR to ~780 nm | p-type: V_D_ = -80 V, V_G_ = -40 V; | Chiral ambipolar phototransistor; static solid-state interfaces. | 0.45 (p-type) and 0.12 (n-type) at 730 nm | 2.1 × 10^10^ (p-type); | — | \|g_R_\| = 0.057 at 635 nm | [14] |
|  |  |  |  | n-type: V_D_ = +80 V, V_G_ = +40 V |  |  | 5.9 × 10^9^ (n-type) |  |  |  |
| Zhu et al., Nat. Commun. 2022 | NTPH-P or SDT chiral acceptor/DPA organic crystal | CPL-selective absorption, donor-acceptor exciton separation, and FET amplification | 488, 556, and 700 nm | V_D_ = -60 V; V_G_ = -10 V | Donor-acceptor heterojunction OFET; fixed solid-state interface. | 0.28 at 556 nm | — | t_r_/t_f_ = 45/46 ms;  ~21 Hz | \|g_ph_\|= 0.24 at 556 nm | [15] |
| Wan et al., Nat. Photon. 2023 | R/S 5011-induced chiral ITIC: PBDBT non-fullerene acceptor blend | Supramolecular excitonic coupling and CPL-selective absorption in the NFA blend | 600 to 1000 nm | self-powered | Self-powered chiral organic photodiode; fixed bulk-heterojunction interface. | — | 1.07 ± 0.05 × 10^13^ at 730 nm | t_r_/t_f_ = 3.48/3.48 μs; ~120 kHz | \|g_ph_\| = 0.113 at -2 V | [16] |
| Song et al., Nature 2023 | Chirally templated PCPDTTBTT/PC_70_BM helical polymer blend | Long-range supramolecular helical ordering with photomultiplication-enhanced electrical readout | Broad visible response | maximum \|g_ph_\| at -1 V | Photomultiplication photodiode array; fixed solid-state junction. | — | 1 × 10^11^ | — | \|g_ph_\| = 1.2 | [17] |
| Bai et al., J. Am. Chem. Soc. 2024, | (R/S-PyEA)Pb_2_Br_6_ chiral 3D perovskite single-crystal microwires | Intrinsic CPL-selective absorption in oriented chiral 3D single crystals | 365 nm | 5 V | Wafer-scale chiral single-crystal array; static crystal/electrode interfaces. | 86.7 | 4.84 × 10^13^ | t_r_/t_f_=428.0/141.6 μs | g_res_ = 0.42 | [18] |
| Yang et al., Nat. Commun. 2025 | Chiral-2D / 3D perovskite heterostructure | Optical spin injection in the 3D absorber followed by CISS-based spin filtering through the chiral-2D layer | 400-760 nm | self-powered | Vertical chiral-2D/3D spin detector; fixed solid-state heterointerface. | 0.0832 | 3.7 × 10^11^ | t_r_/t_f_ = 81/72 μs; ~5 kHz | \|g_current_\| ≈ 0.28 over 400-600 nm | [19] |
| **This work** | **Six-layer R/S cholesteric polymer film+BiVO_4_/RGO/MXene (BRM)** | **Chiral photonic gating followed by self-powered solid-liquid PEC transduction** | **360-830 nm** | **self-powered** | **Modular CP/BRM PEC architecture; dynamic solid-liquid interfacial charge regulation suppresses pinning and noise.** | **0.1108** | **1.06 × 10^11^** | **t_r_/t_f_ = 29.8/30.1 ms (oscilloscope)** | **\|g_Iph_\| = 1.74 at 447 nm; g_Iph_/g_CD_ ≈ 1.8** | **This work** |
| Note: The reported values were obtained under different wavelengths, optical powers, biases, device areas, noise-analysis methods, and definitions of the dissymmetry factor and are therefore provided for representative comparison rather than strict performance ranking. "—" indicates that the corresponding value was not reported or was not directly comparable. | | | | | | | | | | |

Representative CPL detectors based on different chiral layers and signal-transduction architectures are summarized in Table S2^12, 13, 14, 15, 16, 17, 18, 19^. The comparison includes the chiral material, device architecture, CPL-discrimination mechanism, spectral range, operating bias, responsivity, specific detectivity, response time or bandwidth, electrical dissymmetry factor, and key architectural feature. Because the reported values were obtained under different wavelengths, incident powers, applied biases, active areas, and noise-analysis methods, the comparison is intended to illustrate representative architectural and performance characteristics rather than provide a strict ranking. For solid-state CPL detectors employing heterojunction- or photonic-filter/transducer architectures, the relevant semiconductor, heterojunction, dielectric, or electrode interfaces are structurally fixed. In contrast, the present design spatially separates CPL-selective optical modulation from photoelectrochemical transduction by coupling a six-layer CP film with a BRM photoelectrode. The two functional components can be prepared separately through solution-compatible processes and subsequently assembled in a modular manner. More importantly, ion-mediated dynamic charge screening and redistribution at the semiconductor/electrolyte interface alleviate trap-state charging and interfacial pinning, thereby suppressing current fluctuations and reducing noise. Together with self-powered operation and detection across six wavelength windows from 360 to 830 nm, these characteristics define the distinct value of the present solid-liquid architecture relative to conventional fixed-interface devices.

**References**

1.	L. Wang, L. Han, W. Guo*, et al.* Hybrid Dirac semimetal-based photodetector with efficient low-energy photon harvesting. *Light Sci. Appl.* **11**, 53 (2022).

2.	V. Pecunia, T. D. Anthopoulos, A. Armin*, et al.* Guidelines for accurate evaluation of photodetectors based on emerging semiconductor technologies. *Nat. Photon.* **19**, 1178-1188 (2025).

3.	D. Lee, W. Wang, C. Zhou*, et al.* The impact of surface composition on the interfacial energetics and photoelectrochemical properties of BiVO_4_. *Nat. Energy* **6**, 287-294 (2021).

4.	Z. Xie, H. L. Tan, X. Wen*, et al.* The Importance of the Interfacial Contact: Is Reduced Graphene Oxide Always an Enhancer in Photo(Electro)Catalytic Water Oxidation? *ACS Appl. Mater. Interfaces* **11**, 23125-23134 (2019).

5.	J. H. Kim, J. S. Lee. Elaborately Modified BiVO_4_ Photoanodes for Solar Water Splitting. *Adv. Mater.* **31**, 1806938 (2019).

6.	S. Zhou, C. Jiang, J. Han, Y. Mu, J. R. Gong, J. Zhang. High-Performance Self-Powered PEC Photodetectors Based on 2D BiVO_4_/MXene Schottky Junction. *Adv. Funct. Mater.* **35**, 2416922 (2025).

7.	C. G. Ferreira, C. Ros, M. Zhang*, et al.* Sub-Bandgap Photon-to-Current Conversion in Bismuth Vanadate Photoanodes and Its Impact on the Maximum Photocurrent Density Achievable for Water Splitting. *ACS Energy Lett.* **10**, 4332-4341 (2025).

8.	S. Selim, E. Pastor, M. García-Tecedor*, et al.* Impact of Oxygen Vacancy Occupancy on Charge Carrier Dynamics in BiVO4 Photoanodes. *J. Am. Chem. Soc.* **141**, 18791-18798 (2019).

9.	Z. Zhang, J. T. J. C. r. Yates Jr. Band bending in semiconductors: chemical and physical consequences at surfaces and interfaces. *Chem. Rev.* **112**, 5520-5551 (2012).

10.	W. A. Smith, I. D. Sharp, N. C. Strandwitz, J. J. E. Bisquert, E. Science. Interfacial band-edge energetics for solar fuels production. *Energy Environ. Sci.* **8**, 2851-2862 (2015).

11.	J. Li, Y. Long, Z. Hu*, et al.* Kinetic photovoltage along semiconductor-water interfaces. *Nat.Commun.* **12**, 4998 (2021).

12.	P. Grey, S. N. Fernandes, D. Gaspar*, et al.* Field-Effect Transistors on Photonic Cellulose Nanocrystal Solid Electrolyte for Circular Polarized Light Sensing. *Adv. Funct. Mater.* **29**, 1805279 (2019).

13.	C. Chen, L. Gao, W. Gao*, et al.* Circularly polarized light detection using chiral hybrid perovskite. *Nat.Commun.* **10**, 1927 (2019).

14.	L. Zhang, I. Song, J. Ahn*, et al.* π-Extended perylene diimide double-heterohelicenes as ambipolar organic semiconductors for broadband circularly polarized light detection. *Nat.Commun.* **12**, 142 (2021).

15.	D. Zhu, W. Jiang, Z. Ma*, et al.* Organic donor-acceptor heterojunctions for high performance circularly polarized light detection. *Nat.Commun.* **13**, 3454 (2022).

16.	L. Wan, R. Zhang, E. Cho*, et al.* Sensitive near-infrared circularly polarized light detection via non-fullerene acceptor blends. *Nat. Photon.* **17**, 649-655 (2023).

17.	I. Song, J. Ahn, H. Ahn*, et al.* Helical polymers for dissymmetric circularly polarized light imaging. *Nat.* **617**, 92-99 (2023).

18.	J. Bai, H. Wang, J. Ma*, et al.* Wafer-Scale Patterning Integration of Chiral 3D Perovskite Single Crystals toward High-Performance Full-Stokes Polarimeter. *J. Am. Chem. Soc.* **146**, 18771-18780 (2024).

19.	L. Yang, Y. Gao, Z. Wang, L. Yang, M. Shao. Spin detector for panchromatic circularly polarized light detection. *Nat.Commun.* **16**, 4161 (2025).
